# Bacterial genome reconstruction and community profiling in Neotropical *Drosophila*

**DOI:** 10.1038/s41598-026-36282-y

**Published:** 2026-01-29

**Authors:** Maria Alejandra Ulloa, Angela Viviana Serrano, Laura Carolina Camelo, Romain Guyot, Doris Vela, Alejandro Reyes Muñoz

**Affiliations:** 1 Group in Computational Biology and Microbial Ecology, Department of Biological Sciences, Universidad de Los Andes, Bogotá, Colombia; 2 Institut de Recherche Pour Le Développement (IRD), UMR DIADE, CIRAD, Université de Montpellier, 34394 Montpellier, France; 3 Facultad de Ciencias Exactas, Naturales y Ambientales, Laboratorio de Genética Evolutiva, Pontificia Universidad Católica de Ecuador, Quito, Ecuador; 4 Wellcome Sanger Institute, Cambridge, UK

**Keywords:** Neotropical *Drosophila spp*, Metagenome-assembled genomes (MAGs), Microbiota, Gut bacteria, Phylogenetic analysis, Ecology, Ecology, Microbiology

## Abstract

*Drosophila* species serve as key models for microbiota research due to their relatively simple microbial communities. However, microbial diversity and dynamics in Neotropical Andean *Drosophila* remain underexplored. Here we applied shotgun metagenomics to characterize the microbiota of 24 Neotropical *Drosophila* species from Ecuador, reconstructing 64 high-quality bacterial genomes predominantly from Acetobacteraceae and Enterobacterales. Microbial communities were consistently dominated by yeasts, lactic acid bacteria, acetic acid bacteria, and *Wolbachia*. Comparative analyses revealed no strong correlation between host phylogeny and microbial community composition, suggesting environmental factors and microbial interactions shape these communities. Notably, shifts in relative abundances indicate dynamic ecological succession and metabolic cooperation among microbes. These findings expand genomic resources for *Drosophila*-associated bacteria and highlight the complex ecological processes influencing host–microbiota relationships in natural populations.

**Supplementary Information:**

The online version contains supplementary material available at 10.1038/s41598-026-36282-y.

## Introduction

*Drosophila* species, known for their simple microbiota, rapid life cycles, and ease of laboratory cultivation, serve as a robust model for studying host-microbiota interactions^1–3^. Several studies have demonstrated that microbiota play a key role in *Drosophila* health, influencing development, physiology, reproduction, behaviour, and immune system function^4–12^. For instance, the gut microbiota has been shown to promote larval growth under nutrient-limited conditions by modulating host nutrient-sensing pathways^9^. In adult flies, the microbiota can also modulate behaviours such as aggression, potentially via the promotion of octopamine production, with downstream effects on sexual fitness and mating success^13^. These findings underscore the diverse and dynamic roles of the microbiota in shaping host biology across developmental stages.

Given the importance of this relationship, previous analyses have characterised and described the composition of microbiota associated with different *Drosophila* species. These studies concluded that various factors such as nutrient availability, geographic location, and social interactions, influence community composition and structure^14^. Additionally, host-specific factors, including phylogeny, genetics, immune mechanisms, physiology, and vertical transmission, can influence microbial community structure by filtering and selecting for specific taxa^4,6,15^. Taxa such as Enterobacteriaceae, Lactobacillales, and Acetobacteraceae have been repeatedly identified across multiple *Drosophila* species, including *D. hydei, D. elegans, D. virilis,* and *D. melanogaster,* suggesting the existence of a core microbiota^2,16–20^.

Given the close host–microbiome associations, it has been hypothesised that the microbiota may reflect the evolutionary history of their hosts. Supporting this hypothesis, previous studies have found that microbial community clustering mirrors host phylogeny^21,22^. In insects for example, it has been observed in the viromes of Nasonia wasps^23^, and in the gut microbiota of cockroaches and termites Blattodea order^24^. However, evidence for phylosymbiosis in *Drosophila* is mixed, with some studies finding weak support or no clear pattern in both laboratory and wild populations^21^.

In this study, we aimed to address the limited knowledge of the microbiota in Neotropical *Drosophila* species, particularly those endemics to Ecuador. We sampled 24 *Drosophila* species from nine Ecuadorian provinces (Chimborazo, Cotopaxi, El Oro, Imbabura, Loja, Napo, Pichincha, Tungurahua, and Zamora Chinchipe) to characterise their microbiota. We applied a comprehensive bioinformatic workflow using whole-genome shotgun sequencing data to assess microbiota composition, including bacterial and fungal species, and to reconstruct high-quality complete or nearly complete bacterial Metagenome Assembled Genomes (MAGs). Additionally, we investigated whether the microbial community composition and structure reflect the evolutionary history of these *Drosophila* species or whether they are more affected by other environmental forces such as diet.

## Results

### Retrieval of microbial reads from raw *Drosophila* libraries

**Fig. 1:**
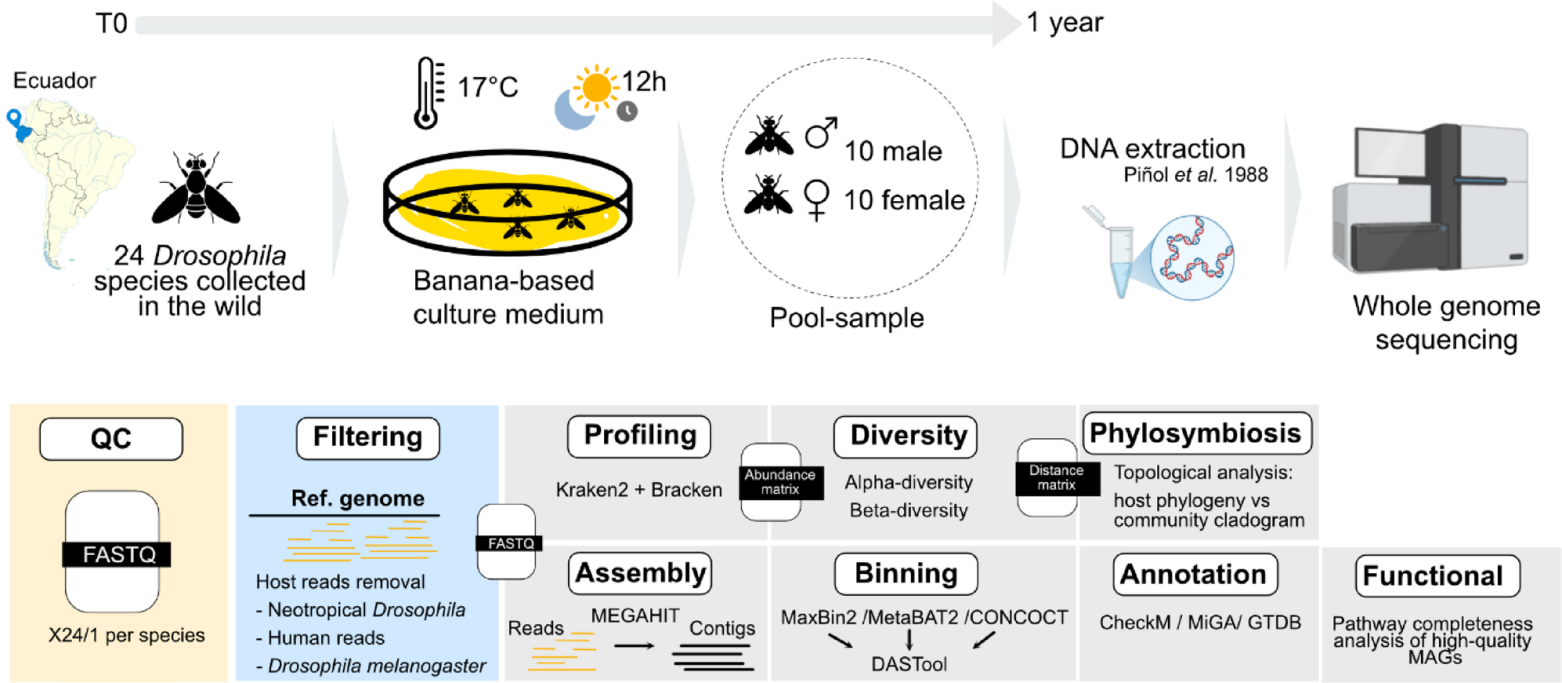
Sample collection and general bioinformatic workflow. *Drosophila* flies from 24 species were collected in the wild and maintained for several generations (up to one year) on a banana-based culture medium under controlled conditions (17 °C, 12 h light/12 h dark photoperiod). For each species, pooled samples consisting of 10 females and 10 males were used for DNA extraction and shotgun metagenomic sequencing. Raw sequencing reads underwent quality control and filtering to remove host and human reads. The resulting microbial reads were used in parallel for two analytical streams: (i) metagenome assembly, binning, genome quality assessment, annotation, and functional analyses; and (ii) taxonomic profiling using Kraken2/Bracken, followed by alpha- and beta-diversity analyses and phylosymbiosis assessment. Some icons in this figure were created with BioRender.com.

Randomly sheared *Drosophila* DNA (Table 1) was extracted from the 24 *Drosophila* species. DNA libraries were sequenced using Illumina paired-end chemistry (see Methods and Fig. 1). The total number of reads per library ranged from 24 to 161 million reads, with an average of 81 ± 33 million sequences (Supplementary Table 1). A read-based analysis revealed a substantial presence of bacterial and fungal sequences, comprising 2% to 22% of the total reads relative to host-derived sequences. An initial K-mer distribution analysis performed on the raw datasets enabled the separation of host and microbial reads. To characterise the microbial composition and diversity, we extracted the microbial fraction from the raw *Drosophila* datasets for downstream analyses (see methods).

**Table 1:** Drosophila species metadata.

Library	Species	Species-group	Province
*Damag*	*D.amaguana*	mesophragmatica	Pichincha
*Dasiri*	*D. asiri*	asiri	Pichincha
*Dcarl*	*D. carlosvilelai*	tripunctata	Pichincha
*Dcash*	*D. cashapamba*	mesophragmatica	Pichincha
*Dchor*	*D. chorlavi*	mesophragmatica	Imbabura
*Dguac*	*D. guacamayos*	not grouped	Napo
*Dguayllabambae*	*D. guayllabambae*	repleta	Chimborazo, El Oro, Imbabura, Loja, Pichincha, Tungurahua
*Dmach*	*D. machachensis*	tripunctata	Pichincha
*Dmeso*	*D. mesophragmatica*	mesophragmatica	Chimborazo, Cotopaxi, Loja, Pichincha, Tungurahua
*Dnam*	*Species B (nambillo)*	guarani	Pichincha
*Dneoama*	*D. neoamaguana*	mesophragmatica	Napo
*Dneoasiri*	*D. neoasiri*	asiri	Napo
*Dnina*	*D. ninarumi*	tripunctata	Pichincha
*Dpapallacta*	*D. papallacta*	not grouped	Pichincha
*Dpaso*	*D. pasochoensis*	tripunctata	Pichincha
*Dquitensis*	*D. quitensis*	guarani	Pichincha
*Drucux*	*D. rucux*	mesophragmatica	Pichincha
*Dsang*	*D. chamanapamba*	not grouped	Tungurahua
*Dshunku*	*D. shunku*	not grouped	Loja
*Dtars*	*D. tarsata*	annulimana	Napo, Pichincha
*Dulba*	*D. ulba*	not grouped	Zamora Chinchipe
*Durcu*	*D. urcu*	tripunctata	Pichincha
*Dvale*	*D. valenteae*	guarani	Pichincha
*Dyanayuyu*	*D. yanayuyu*	mesophragmatica	Pichincha

### Read-based taxonomic profiling and diversity

To assess the taxonomic composition of the *Drosophila* microbiota, we used Kraken2^25^ and Bracken^26^ for metagenomic classification and abundance estimation. Taxa with relative abundance below 1% were excluded (Supplementary Table 2). Analysis of bacterial communities revealed that the *Drosophila* microbiota was primarily composed of *Acetobacter* and *Gluconobacter* species (order Rhodospirillales) (Fig. 1A). Additional taxa include members of Enterobacteriales, such as *Morganella* and *Providencia*, and Lactobacillales, such as *Levilactobacillus*.

**Fig. 2:**
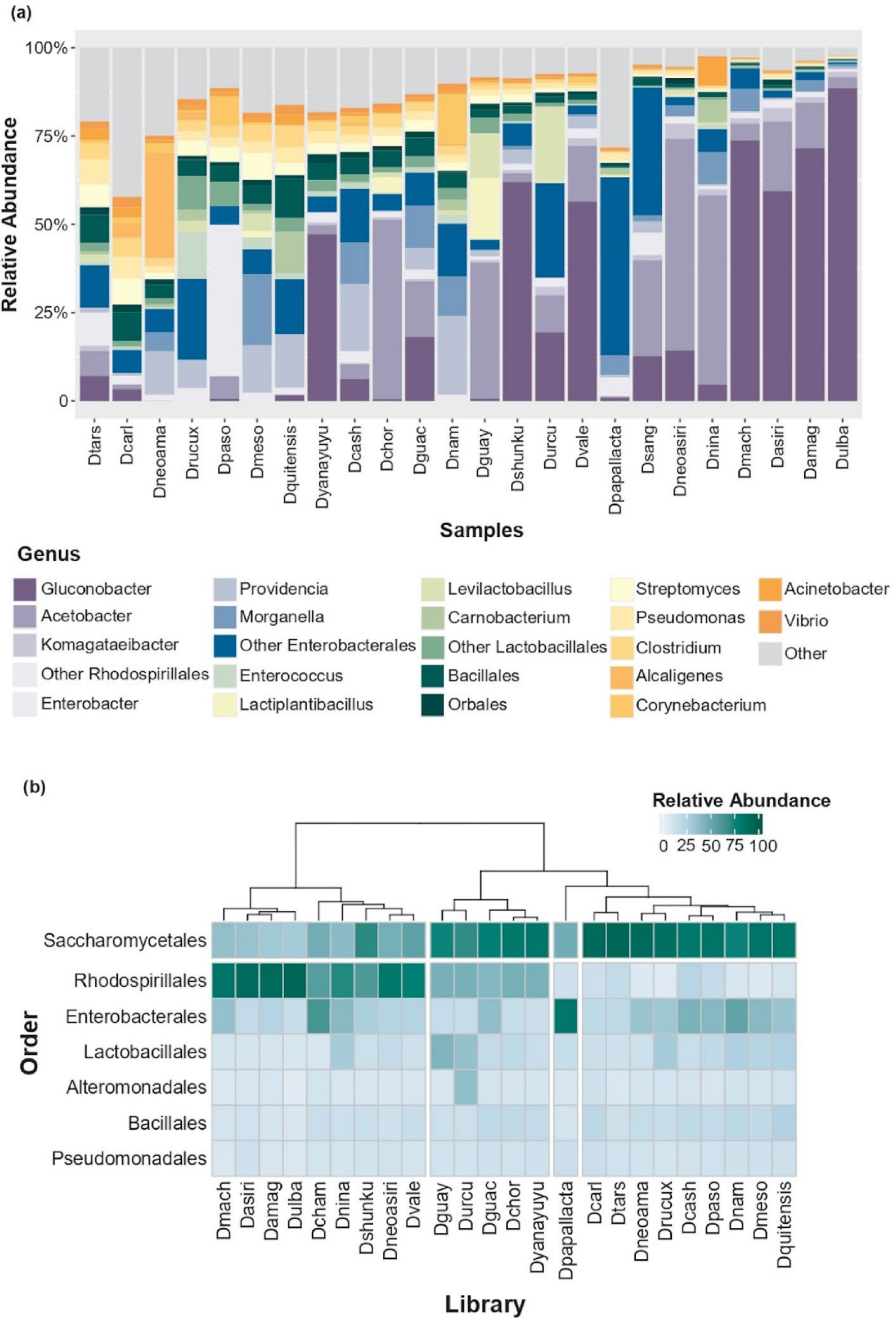
Taxonomic classification of metagenomic reads. Taxonomic profiling was performed using Kraken2, with relative abundances estimated via Bracken. (**a**) Relative abundance of bacterial taxa at the genus level. Colours correspond to the taxonomic order to which each genus belongs, providing a visual distinction among species-groups (very related taxa). Taxa with a relative abundance below 1% are grouped under the “others” category. (**b**) Heatmap showing the relative abundance of bacterial and yeast taxa at the order level. Samples are arranged horizontally based on their Bray–Curtis dissimilarity index, revealing similarities and differences between microbiota. Yeast taxa are displayed above bacterial groups to highlight the distinct contributions of both microbial types.

We observed a pattern where Rhodospirillales (mainly *Acetobacter* and *Gluconobacter*) tended to be more abundant in samples where Enterobacteriales and Lactobacillales were less prominent, and vice versa suggesting the dominance of one taxonomical group over the others. This indicates the presence of at least two distinct clusters: one dominated by Rhodospirillales and the other by Enterobacteriales (Fig. 2A). The inclusion of eukaryotic reads in the abundance estimation revealed that yeasts from the order Saccharomycetales were the most prevalent eukaryotic taxa across samples (Fig. 2B). Notably, samples with high Rhodospirillales abundance also showed increased yeast abundance compared to samples dominated by Enterobacteriales (Wilcoxon rank sum exact test, *p*-value = 0.001199, Supplementary Fig. S1A).

We also detected *Wolbachia* sequences in the species *D. carlosvilelai* (1.19% of total reads) and *D. ninarumi* (6.99% of total reads) (Supplementary Table 2). However, these sequences were excluded from the bacterial abundance estimation, as *Wolbachia* primarily resides in the gonads and is not part of the gut microbiota^27^.

### Beta and Alpha diversity analysis of *Drosophila* bacterial microbiota: influence of species classification, environment, and taxa abundance

**Fig. 3:**
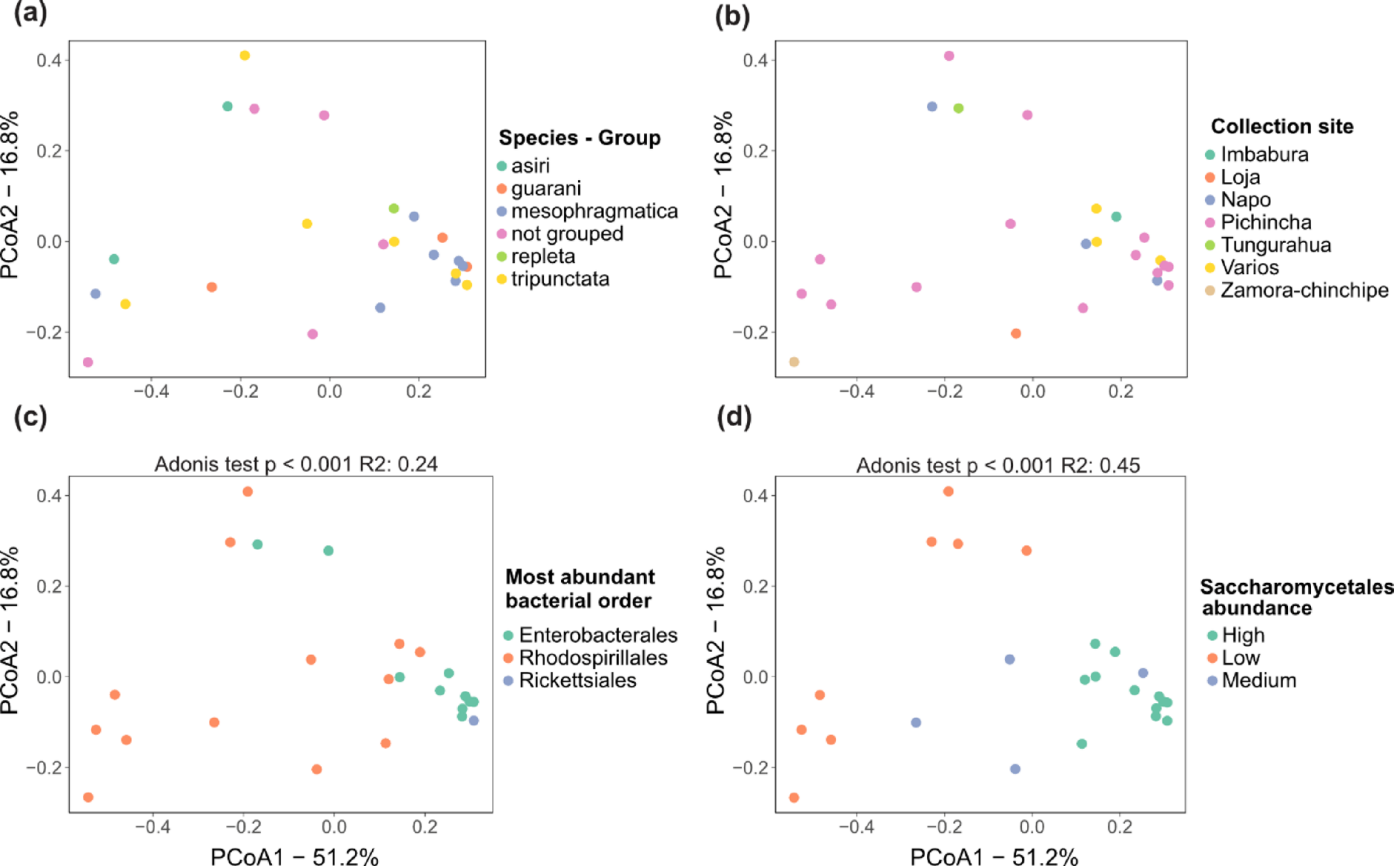
Principal Coordinate Analysis (PcoA) of microbiota based on the relative abundance of yeast and bacterial reads (excluding the endosymbiont *Wolbachia*), calculated using Bray–Curtis’s dissimilarity. (**A**–**D**) display PCoA results coloured according to different categorical variables. **(A)** Grouping by species-groups, also defined as a group of closely related species with often unclear boundaries due to recent evolutionary divergence; **(B)** Collection site of samples; **(C)** Most abundant bacterial order, defined as those orders representing more than 40% of the total relative abundance of reads; **(D)** Abundance of Saccharomycetales yeast, categorised as follows: high (relative abundance > 60%), medium (30–60%), and low (relative abundance < 30%). The results of the Adonis test are indicated for comparisons with significant *p-value* < 0.001.

To evaluate the factors influencing *Drosophila* microbiota structure and diversity, we performed a permutational multivariate analysis of variance (PERMANOVA) using dissimilarity matrices from bacterial reads^28^. We tested whether factors such as species relatedness (species-group taxonomical classifications), collection site, or abundance of specific taxa significantly influenced the bacterial microbiota structure.The first two principal coordinates explained more than 60% of the variation in the PCoA based on Bray–Curtis dissimilarity matrix. Sample distribution along both axes showed no significant association with group-species or collection site (Fig. 3A, B). However, the relative abundance of Saccharomycetales yeasts (PERMANOVA: P < 0.001, R2 = 0.45) and the ratio between Rhodospirillales and Enterobacteriales (PERMANOVA P < 0.001, R2 = 0.24) were significantly associated with microbiota structure (Fig. 3C, D).

For Jaccard distances, we observed high dispersion and no clear clustering pattern (Supplementary Fig. 1). Alpha diversity was assessed using Shannon’s diversity index^29^ and Chao1^30^. No significant differences were found among species groups (Shannon: *p* = 0.802; CHAO1: *p* = 0.638; Kruskal–Wallis test, *p* < 0.05) (Supplementary Fig. 2).

### Reconstruction of *Drosophila*-associated bacterial genomes

**Fig. 4:**
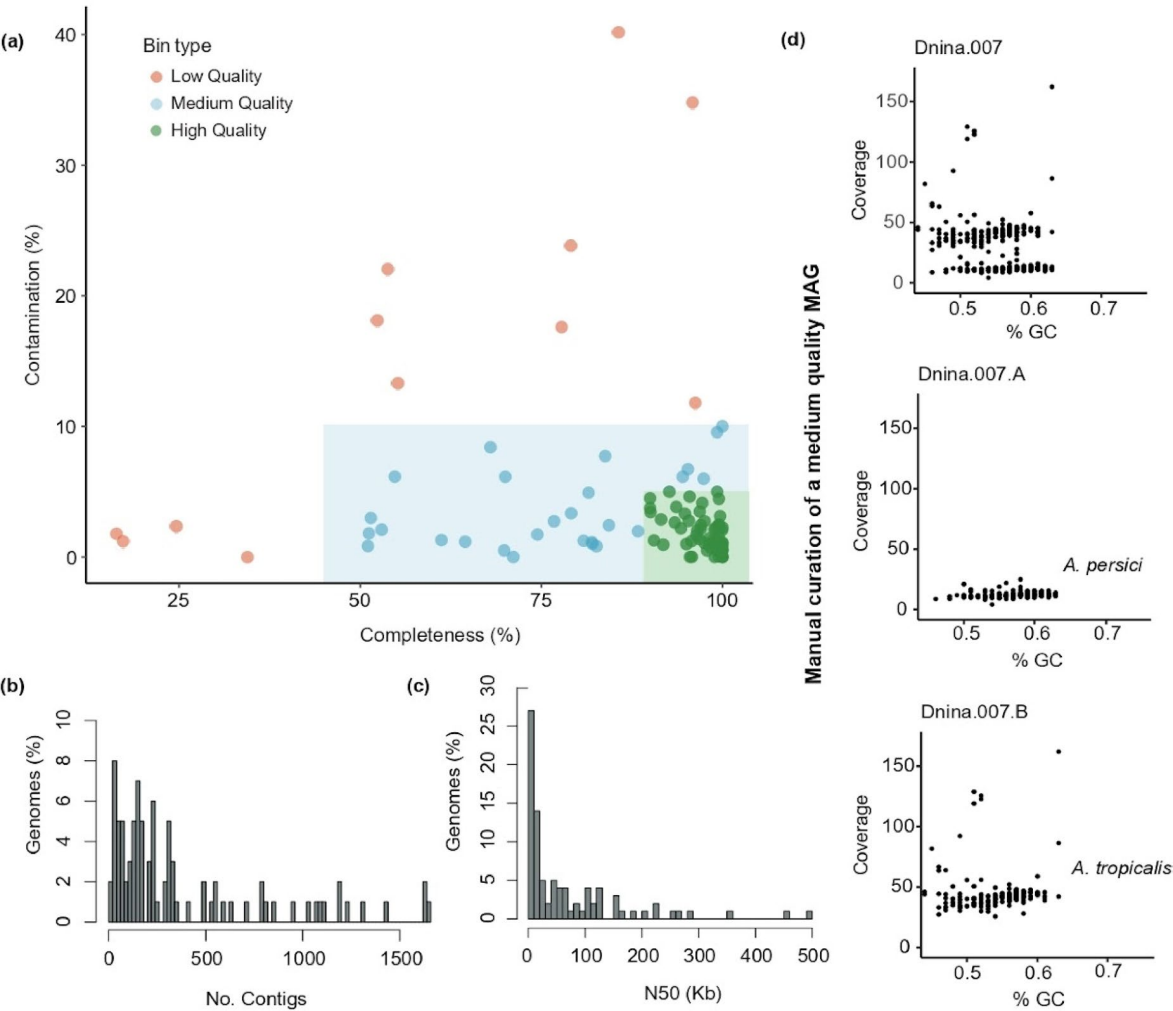
Genomic features of draft genomes and manual curation. **(a)** Assessment of completeness and contamination in reconstructed genomes: 64 “high-quality” bins (completeness > 90% and contamination < 5%); 27 “medium quality” bins (completeness ≥ 50% and contamination < 10%); and 12 “low-quality” bins (completeness < 50% and contamination < 10%). **(b)** Number of contigs for medium and high-quality bins, with a minimum of 13 contigs and a maximum of 1641 contigs observed. **(c)** N50 values for medium and high-quality bins, illustrating the contiguity of the assemblies. **(d)** Manual curation of the *Dnina.007* bin revealed distinct coverage patterns in the reads, suggesting the presence of two different populations within the same bin. Upon separating these into two distinct bins and conducting taxonomic assignment, the newly identified metagenome-assembled genomes (MAGs) correspond to *Acetobacter persici* (Dnina.007.A) and *Acetobacter tropicalis* (Dnina.007.B).

To reconstruct bacterial genomes from the *Drosophila* microbiota, we first removed fungal sequences, assembled the reads into contigs, and binned them into metagenome-assembled genomes (MAGs). We used a single-assembly strategy with multiple binning tools and aggregated results using DAS-Tool (see Methods).We recovered 64 MAGs with < 5% contamination, assigned to the following bacterial classes: Alphaproteobacteria (34), Gammaproteobacteria (21), Bacteroidia (7), Bacilli (1), and Betaproteobacteria (1) (Supplementary Table 3). For high-quality MAGs, the median N50 was 28 kb (Fig. 4C), while the median number of contigs was 134; medium-quality MAGs showed a higher median number of contigs (212; Fig. 4B).

To improve MAG quality, we curated medium- and high-quality bins by analysing contig coverage and GC content. For the medium-quality bin Dnina.007 we identified two distinct contig populations with similar GC content (~ 54–55%) but different coverages (12.46 ± 2.33 and 43.21 ± 14.00, respectively) (Fig. 4D). We manually separated these into two new bins: Dnina.007.A (*Acetobacter persici*) and Dnina.007.B (*Acetobacter tropicalis*).

### Taxonomic assignment of metagenome-assembled genome

Taxonomic assignment of MAGs was performed with MIGA^31^ and GTDB-Tk^32^. Based on consensus annotations from both tools for high-quality bins, the predominant phylogroups at the family and genus levels were: Acetobacteraceae: *Acetobacter* (n = 15), *Gluconobacter* (n = 13); Enterobacteriaceae: *Providencia* (n = 4), *Cedecea* (n = 7); Morganellaceae: *Morganella* (n = 6); and Dysgonomonadaceae: *Dysgonomonas* (n = 7). In total, 37 MAGs were assigned to the species level (Supplementary Table 3). The most frequently identified species were *Acetobacter thailandicus* (n = 10), *Gluconobacter kondonii* (n = 6), *Gluconobacter wancherniae* (n = 4), and *Providencia sp003533305* (n = 3). Unique species were detected in specific *Drosophila* species, including *Levilactobacillus brevis*, *Alcaligenes pakistanensis*, and *Acinetobacter guillouiae*.

We reconstructed MAGs for all major taxa identified in the community profiling, except for the *D. quitensis* and *D. mesophragmatica* libraries, where no MAGs were recovered (Supplementary Table 1). Most reconstructed MAGs corresponded to highly abundant taxa, particularly members of the *Acetobacter* genus, including *A. thailandicus*, *A. tropicalis*, *A. persici*, and *A. sicerare*. Among *Gluconobacter* species, *G. kondonii* was the most common, followed by *G. wancherniae*, *G. cerinus*, and *Gluconobacter* sp014132155. For the order *Lactobacillales*, only one genome—*Levilactobacillus brevis*—was reconstructed, showing 98.61% ANI to GCF_001433855.1. However, no MAGs were reconstructed for the *Lactobacillales* or Bacillales groups from the *D. guayllabambae* and *D. quitensis* libraries, despite their high estimated relative abundances (33% and 10%, respectively; Fig. 2A, B).

To assess whether this discrepancy reflected taxonomic misclassification or limitations in genome reconstruction, we performed a targeted read- and contig-level analysis of the *D. quitensis* dataset. We extracted 14,859 reads classified as *Carnobacterium* (formerly *Lactobacillus*)—one of the most abundant *Bacillales* genera in this library—and confirmed their taxonomic identity by BLASTn searches against the NCBI nt database, which predominantly matched *Carnobacterium maltaromaticum*. Mapping these reads to a reference genome (GCF_949790605.1) using Bowtie2 resulted in a high alignment rate (95.7%) and relatively uniform coverage across the genome, with an average depth of ~ 40 × (Supplementary Fig. 2C). In contrast, mapping against the MEGAHIT assembly showed markedly reduced and uneven coverage distributed across 260 contigs, accompanied by low completeness estimates based on CheckM (Supplementary Fig. 2D). These results indicate that, despite the presence of abundant species-specific reads, genome reconstruction was hindered by fragmented assembly and uneven coverage rather than by incorrect taxonomic classification.

### All-vs-all ANI estimated for high-quality MAGs

To assess relatedness among genomes, we performed an all-vs-all average nucleotide identity (ANI) analysis of all high-quality MAGs. The results revealed at least seven major clusters with ANI values ≥ 95% (Supplementary Fig. 3). The largest cluster, comprising ten genomes, had an average ANI of 99.01% and was identified as *Acetobacter thailandicus*, confirming conspecificity. *Gluconobacter* genomes formed another distinct cluster, with an average ANI of 85.23%. Within this cluster, subclusters corresponded to *G. kondonii* and *G. wancherniae.* MAGs from *Cedecea, Morganella,* and *Dysgonomonas* also formed well-defined clusters, indicating genetic coherence within these genera. (Supplementary Fig. 3). ANI-based clustering supported the accuracy of taxonomic annotations. However, ANI values > 79% among *Acetobacter*, *Gluconobacter*, and *Morganella* MAGs suggest potential taxonomic overlap.

### Functional characterization of metagenome-assembled genomes

Functional characterization of the metagenome-assembled genomes (MAGs) using the COG and KEGG databases revealed distinct functional profiles that align with their taxonomic annotations. This analysis identified both shared metabolic capabilities and specialized adaptations within the bacterial community.

**Fig. 5:**
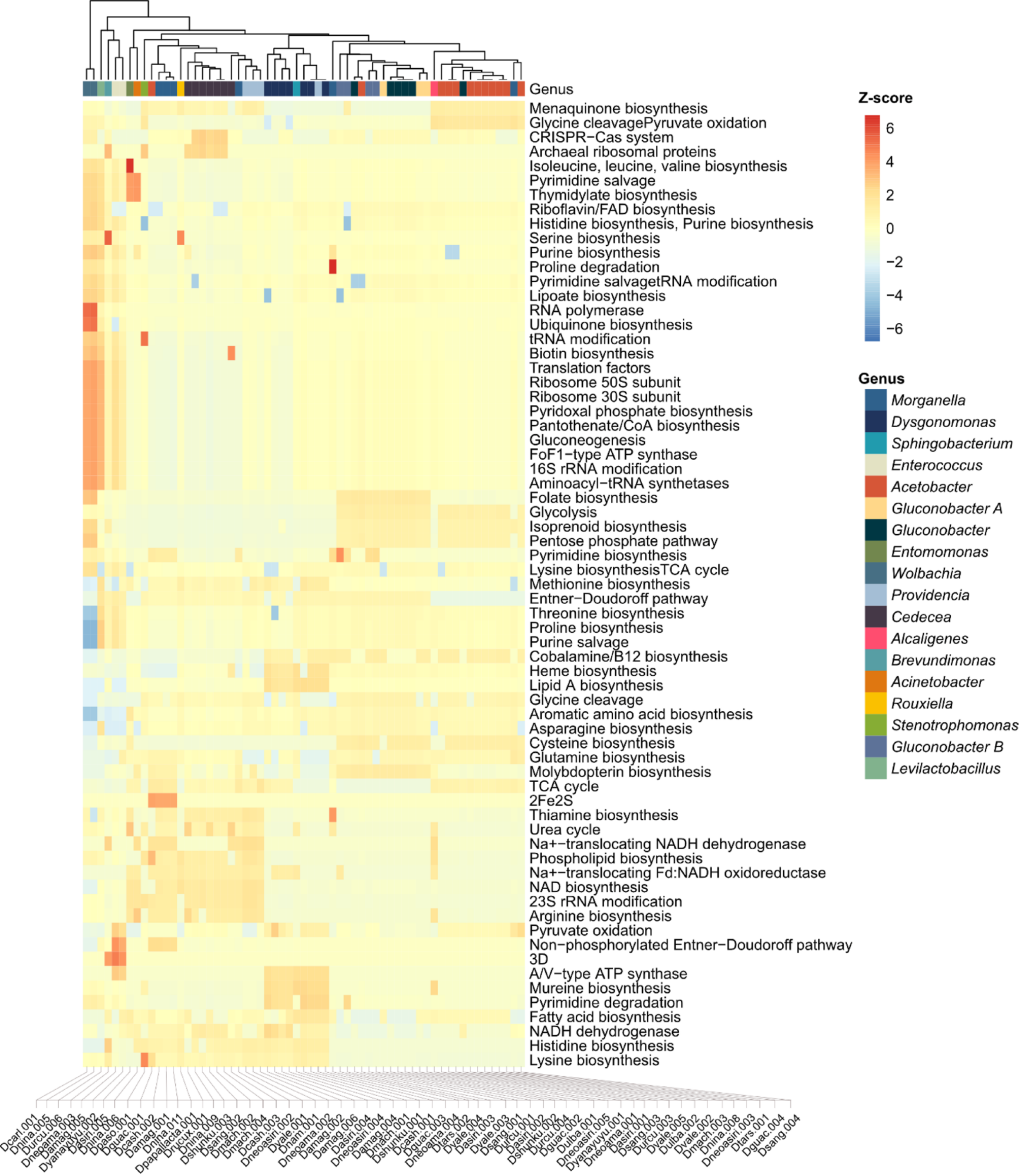
Heatmap of relative abundance of COG functional categories across genomes. Genes were predicted using Prodigal and annotated with DIAMOND against the NCBI COG database. Functional category counts were centered and scaled across genomes to compute row-wise z-scores. Only the most abundant metabolism-related categories are shown. Clustering was performed using Euclidean distance and complete linkage for both rows and columns. Higher z-scores (red) indicate above-average representation of a category in each genome, while lower scores (blue) indicate below-average representation.

The primary carbohydrate metabolism pathways varied among the identified taxa. A commonality across *Dysgonomonas*, *Cedecea*, *Morganella*, *Providencia*, and *Acinetobacter* was the functional potential for the Entner-Doudoroff (ED) pathway for glucose catabolism, suggesting a prevalent strategy for carbohydrate utilization. In contrast, while members of the Acetobacteraceae family, specifically *Acetobacter* and *Gluconobacter*, also possess glycolysis genes, *Gluconobacter* uniquely lacks a complete TCA cycle, relying predominantly on the ED pathway for glucose processing (Fig. 5). Furthermore, the presence of AD/FMN-containing lactate dehydrogenase/glycolate oxidase (GlcD) in Acetobacteraceae and lactate dehydrogenase (LDH) and acetate kinase (AckA) in *Lactobacillus brevis* and *Cedecea* highlights the importance of lactate and acetate interconversion, indicating the generation and consumption of key fermentation products (Supplementary Fig. 4).

Diverse strategies for energy production and redox balance were evident across the MAGs (Supplementary Fig. 4). *Dysgonomonas* exhibited a functional signature for anaerobic metabolism, characterized by pyruvate:ferredoxin oxidoreductase (PorG, PorA, PorB) for acetyl-CoA formation, phosphotransacetylase (Pta) for acetate fermentation, and NuoI, collectively indicating robust energy generation in anoxic or microaerobic conditions. *Stenotrophomonas sp.* displayed oxidative stress tolerance mechanisms, including *trxA*, and *grxD*, coupled with genes for redox-driven cytochrome c biogenesis (nrfF), suggesting adaptation to fluctuating oxygen levels. *Brevundimonas sp.* demonstrated versatile respiratory capabilities, supported by sulfur metabolism-related proteins (DsrC family, TusE) and menaquinone biosynthesis genes, allowing for energy conservation under varied redox states.

Regarding the biosynthesis of essential cofactors, vitamins, and amino acids, significant functional diversity was observed. Genomes belonging to *Wolbachia*, *Enterococcus*, and *Acetobacteraceae* generally encoded comprehensive pathways for the biosynthesis of multiple amino acids (e.g., histidine, serine, isoleucine, leucine, valine) and nucleotides (purine, pyrimidine, thymidylate). These groups also possessed broad capabilities for synthesizing crucial cofactors and vitamins, including folate, isoprenoids, ubiquinone, biotin, and pantothenate/CoA. Importantly, *Wolbachia* was identified as a notable exception, lacking the genetic machinery for cobalamin (Vitamin B12) biosynthesis (Fig. 5).

In terms of information processing and unique functional adaptations, the MAGs revealed regulatory systems and specialized metabolic tools (See Supplementary Fig. 4). *Stenotrophomonas sp.* exhibited an elaborate regulatory network, shown by numerous c-di-GMP signaling proteins (GGDEF, EAL, HD-GYP domains) and a rich repertoire of two-component systems (SpoIIM, BaeS-OmpA), indicative of tuned environmental sensing and lifestyle transitions. This bacterium also showed specialized capabilities for plant-derived carbohydrate degradation (pectate lyase, cellobiose phosphorylase) and surface attachment (type IV pilus). The *Rouxiella* genome encoded factors associated with potential virulence and microbial competition, including the invasion protein IalB and Type VI Secretion System (T6SS) components (e.g., VgrG protein), along with polysaccharide-degrading enzymes such as lysozyme family proteins (ZliS) and chitinases (GH18 family). These features suggest active host interaction, immune evasion, antagonism against competitors, and the ability to exploit complex polymeric substrates. *Brevundimonas sp.* possessed efficient nutrient scavenging mechanisms (BtuB, FepA, TonB-dependent transporters). The *Dysgonomonas* genome demonstrated extensive transport systems for essential ions and nutrients (e.g., iron, cobalamin), alongside multidrug efflux pumps and antibiotic resistance genes.

.

### Placement of MAGs on reference phylogenetic trees

**Fig. 6:**
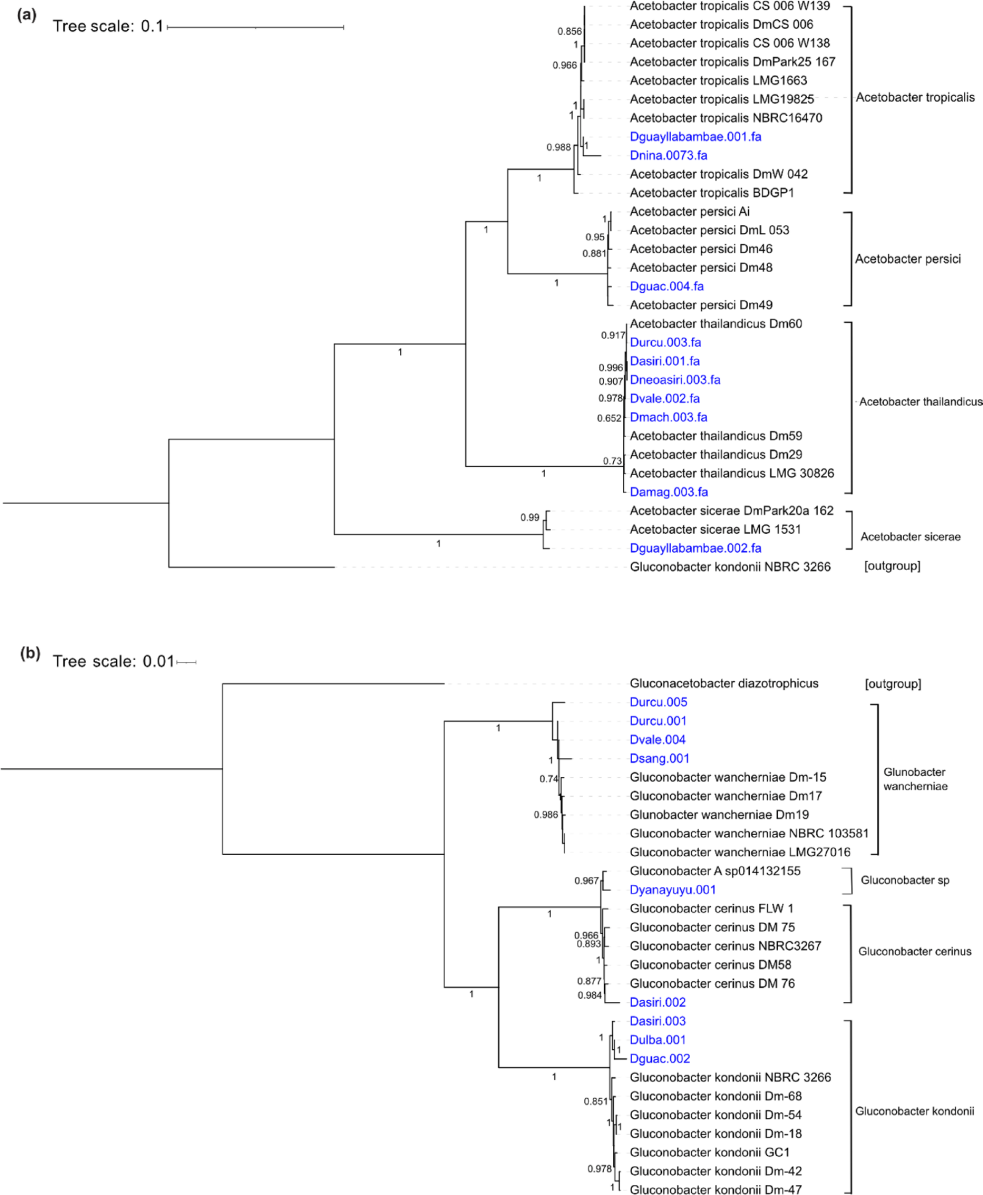
Phylogenetic placement of high-quality MAGs. Genes were predicted using Prodigal, and essential genes shared among all genomes were concatenated for analysis. The phylogenetic tree was constructed using FastTree, with visualization performed in iTOL. Metagenomically reconstructed MAGs are represented in blue, while reference genomes are shown in black. Bootstrap values greater than 0.6 are indicated. (**A**) Phylogenetic tree for the genus *Acetobacter*, based on 95 shared essential genes. (**B**) Phylogenetic tree for the genus *Gluconobacter*, based on 103 shared essential genes.

To further assess the quality of the assembled bacterial genomes, we evaluated the phylogenetic relationships of the recovered high-quality alongside reference genomes from the NCBI RefSeq database (Supplementary table 4). We constructed two maximum likelihood phylogenetic trees based on 95 and 103 essential genes for the genus *Acetobacter* and *Gluconobacter*, respectively. All high-quality MAGs were placed within the same clades as their corresponding NCBI reference genomes, supporting the accuracy of the assembly process (Fig. 6A, B). The *Acetobacter* tree was rooted using *Gluconobacter kondonii* (NCBI RefSeq GCF_002723995.1) as the outgroup. Distinct clades were recovered for *A. thailandicus*, *A. tropicalis*, *A. sicerae*, and *A. persici*. Among the 20 reference genomes used, 14 were isolated from *Drosophila melanogaster* samples (Supplementary Table 3). For the *Gluconobacter* tree, we used *Gluconacetobacter diazotrophicus* (NCBI RefSeq GCF_000021325.1) as the outgroup. The tree revealed four distinct clades corresponding to *G. kondonii*, *G. cerinus*, *G. wancherniae*, and *Gluconobacter* sp014132155. MAG placement was consistent with the taxonomic annotations, and 12 of the 18 reference genomes used were derived from *Drosophila* samples.

### Microbial similarity does not correlate with host evolutionary relatedness

**Fig. 7:**
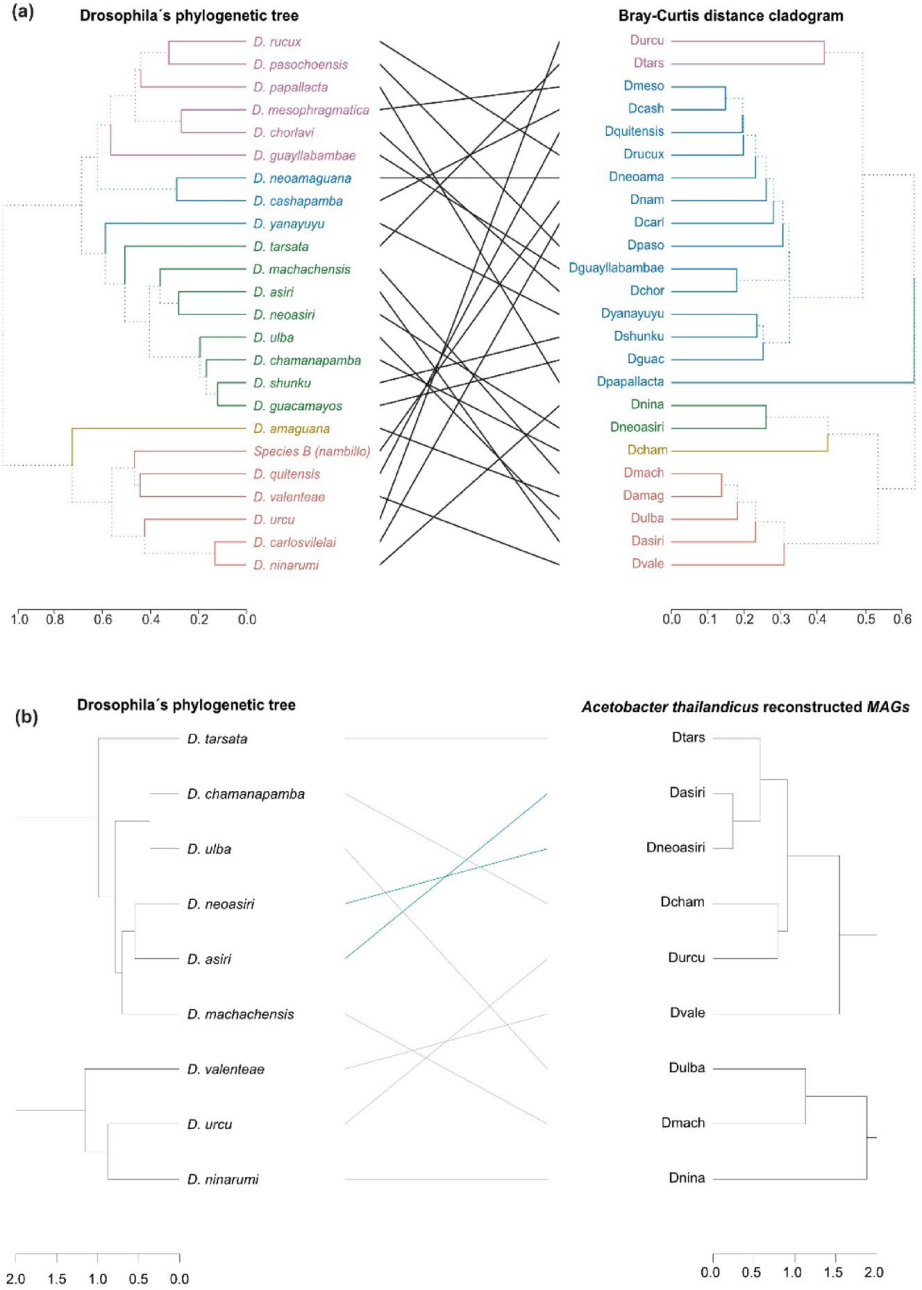
Topological congruency tests comparing host phylogenetic tree and the microbiome dendrogram. (**a**) *Drosophila’s* phylogenetic tree compared to the microbiota dendrogram. The *Drosophila’s* phylogeny was generated using Busco (V5) and the odb10 lineage for Diptera. A super matrix with 106 genes was generated to create the tree. The microbial community dendrogram was generated from the Bray–Curtis dissimilarity matrix calculation. The *Dendextend* package was used to make the visualisation. We used the *TreeDist* package to calculate the similarity using the Generalized Robinson-Foulds metric, mainly calculated the Mutual Clustering Information (MCI) index between both trees and the Clustering Information Distance (CID) was normalised to be bounded for values between 0 and 1 (1 = zero similarity). (**b**) On the right, we display the phylogenetic tree for *Acetobacter thailandicus* reconstructed MAGs, and on the left, the phylogenetic tree of the associated species from where the MAG was reconstructed.

Phylosymbiosis is the hypothesis that closely related host species harbor similar microbiota^21^. Here, we compare the topology of phylogenetic trees and cladograms: one derived from the phylogeny of 24 *Drosophila* genomes (provided by collaborators at IRD and PUCE), and the latter based on Bray–Curtis’s dissimilarity of microbiota. The comparison yielded a generalized Robinson-Foulds score of 0.84^33^, indicating low tree topology similarity (Fig. 7A).

To further assess species-specific relationships, we focused on *Acetobacter thailandicus* MAGs reconstructed from the following *Drosophila* species: *D. asiri*, *D. chamanapamba*, *D. machachensis*, *D. neoasiri*, *D. ninarumi*, *D. tarsata*, *D. valenteae*, *D. ulba*, and *D. urcu*. We compared the phylogenetic tree of these *Drosophila* species with the tree derived from the corresponding *A. thailandicus* MAGs. This comparison resulted in a Robinson–Foulds distance of 0.86 and a Matching Cluster score of 0.6, indicating limited congruence between host phylogeny and *A. thailandicus* genomic relatedness **(**Fig. 7B**)**.

## Discussion

This study provides a metagenomic characterisation of the microbiota associated with 24 Neotropical *Drosophila* species from Ecuador. We observed a relatively consistent microbial composition across species, dominated by yeasts, with the most abundant taxa being Saccharomycetales, and bacteria from the orders Rhodospirillales (acetic acid bacteria, AAB), Enterobacterales, Lactobacillales (lactic acid bacteria, LAB), and the endosymbiont *Wolbachia*. These findings are consistent with previous reports of different *Drosophila* species describing a conserved microbiota structure composed by a set of stable colonizers^2,14,34,35^.

The binning and refinement strategy used in this study enabled the reconstruction of 64 high-quality bacterial genomes. However, for some taxa with high relative abundance based on Kraken2 profiling, metagenome-assembled genomes (MAGs) could not be recovered. This absence is unlikely to reflect erroneous taxonomic classification, as read-level mapping confirmed the presence of abundant sequences with high coverage across reference genomes. Instead, we suspect that genome reconstruction was hindered by assembly fragmentation and uneven coverage, likely driven by high strain similarity within libraries and the presence of closely related taxa. Nevertheless, the high-quality genome assemblies represent a valuable addition to the public resources available for *Drosophila*-associated microbiota, expanding the catalogue of reference-quality genomes for future comparative, evolutionary, and functional analyses. Several of these genomes showed high similarity to published reference genomes from *D. melanogaster* and *D. suzukii,* suggesting that certain bacterial taxa persist across diverse *Drosophila* lineages. Moreover, the genome of *Acetobacter thailandicus* was consistently reconstructed across multiple host species, indicating its widespread presence across the genus.

Given these patterns, we investigated whether microbiota structure mirrors host evolutionary relationships. While previous studies reported an influence of host phylogeny on microbial composition in *D. melanogaster*^21^. Our analysis, based on matrix correlation between Bray–Curtis’s dissimilarities and host phylogenetic distances, as well as topological comparisons of dendrograms, did not reveal a significant association between host relatedness and microbiota structure. These findings suggest that, within this population of *Drosophila*, the host phylogeny does not play a major role in shaping microbial community composition. One limitation of this analysis is that all flies were maintained under standardized laboratory conditions for several generations prior to DNA extraction, a process that may attenuate natural host-specific microbial associations. Under such conditions, shared environmental exposure and diet likely impose strong ecological constraints, favoring taxa that are metabolically adapted to fermentative and sugar-rich environments, such as *Acetobacter* and *Lactobacillus*^14^. Consequently, the observed microbiota structure likely reflects the combined effects of microbial ecology and environmental filtering rather than co-evolutionary history alone.

The convergence in microbiota structure across host species was accompanied by consistent variation in the relative abundance of dominant microbial taxa, particularly yeasts, acetic acid bacteria (AAB), lactic acid bacteria (LAB), and Enterobacterales. Notably, AAB were nearly absent in communities dominated by yeasts, whereas LAB and Enterobacterales fluctuated in tandem with yeast abundance. These patterns suggest environmentally driven shifts in community structure, potentially linked to nutrient availability and the accumulation of fermentation-derived metabolites. Such dynamics are consistent with a model of environmental filtering under standardized laboratory conditions, which may reduce host-specific microbial signatures and contribute to the observed convergence in microbiota composition across Drosophila species.

Functional analyses of reconstructed genomes provide mechanistic support for this interpretation. Genomes from *Acetobacter* and *Gluconobacter* encoded key enzymes involved in lactate, ethanol, and acetate metabolism, including D-lactate dehydrogenase, pyruvate dehydrogenase, and acetyl-CoA synthetase, indicating a genetic capacity to exploit fermentation-derived substrates. Although we cannot confirm that these interactions occur in vivo without targeted metabolic assays or dietary manipulations, similar cross-feeding interactions have been well documented in *Drosophila*-associated microbes. In particular, cooperative metabolic interactions between *Lactiplantibacillus plantarum* and *Acetobacter pomorum* have been shown to influence host physiology and microbial persistence under nutrient-limited conditions^6,8,19,34,36,37^. Furthermore, prior studies have described a complex ecological network within the *Drosophila* microbiota, including mutualism, parasitism, and amensalism, that facilitates both cross-feeding and host-mediated microbial recruitment^7,36–39^.

Comparable successional dynamics are also observed in well-characterized fermentation systems, where LAB initially dominate sugar-rich environments and are subsequently replaced by AAB as ethanol and lactate accumulate and oxygen availability increases^38–42^. In these systems, AAB utilize metabolites produced by yeast and LAB while generating acidic and oxidative conditions that inhibit LAB growth. Together, these ecological precedents support the hypothesis that similar metabolically mediated succession and cross-feeding interactions may shape *Drosophila*-associated microbial communities, particularly in fermenting or sugar-rich environments that favor both LAB and AAB.

In addition to microbial interactions, host physiology may further modulate community assembly by shaping available niches within the gut. Specific regions of the *Drosophila* foregut provide selective binding sites for particular bacterial strains, and early colonization by *Lactobacillus* species has been shown to induce structural changes in the gut epithelium that facilitate subsequent colonization by *Acetobacter*, a process referred to as facilitation^43^. Rather than imposing strict host specificity, such host-mediated effects may act in concert with microbial metabolic interactions to stabilize certain community configurations under shared environmental conditions.

Functional genomic analyses further support the view that dominant members of the *Drosophila*-associated microbiota are metabolically adapted to dynamic, fermentation-influenced environments. The prevalence of the Entner–Doudoroff pathway and the widespread presence of enzymes involved in lactate and acetate metabolism suggest an ability to exploit fermentation-derived substrates under fluctuating redox conditions typical of sugar-rich insect diets. In addition, several taxa exhibited extensive biosynthetic capacities for amino acids, nucleotides, and vitamins, raising the possibility of nutritional contributions to the host. Other specialized functions, including carbohydrate degradation, biofilm formation, secretion systems, and diverse transport and efflux mechanisms, may facilitate colonization, competition, and resilience to host and environment-derived stressors. While the in vivo relevance of these traits remains to be experimentally validated, together they define a coherent set of functional adaptations consistent with ecological persistence in the *Drosophila* gut environment.

In summary, this study expands the genomic and taxonomic landscape of *Drosophila*-associated microbiota by providing a metagenomic characterization across 24 Neotropical species and reconstructing a diverse set of high-quality bacterial genomes. Despite the phylogenetic breadth of hosts examined, we found no strong evidence for phylosymbiosis, suggesting that host evolutionary history alone does not dictate microbiota composition under the conditions studied. Instead, our results point to a model in which environmental filtering, metabolically mediated microbial interactions, and host-driven niche modulation jointly shape community structure. The recurrent presence of taxa such as *Acetobacter* and *Lactobacillus* across hosts, together with functional signatures consistent with cross-feeding and successional dynamics, highlights the importance of ecological processes over strict host specificity. Future studies integrating species-level resolution, experimental manipulation of diet and microbial communities, and analyses of natural populations will be essential to disentangle the relative contributions of environment, host physiology, and microbial interactions in shaping host–microbe associations.

## Methods and materials

### Sample collection and sequencing

Twenty-four *Drosophila* species were collected from nine Ecuadorian provinces (Chimborazo, Cotopaxi, El Oro, Imbabura, Loja, Napo, Pichincha, Tungurahua, and Zamora Chinchipe). Flies were maintained in a banana-based culture medium^44^ under controlled conditions at 17 °C temperature with a 12-h photoperiod for a year. For the DNA extraction, ten females and ten males per species were pooled. DNA was extracted following the protocol described by Piñol et al*.*^45^. Truseq DNA PCR-free libraries with an average insert size of 850 bp were prepared and sequenced on the HiSeq2000 platform (2 × 100 bp, Macrogen).

### Library filtering and clean-up

Adapter removal was performed using Trimmomatic (v.0.39)^46^ with default parameters. As all reads had Phred scores > 30, no quality trimming was required. Read quality was assessed using FastQC (v.0.11.7)^47^. Host-derived and other non-bacterial reads were removed using BMTagger(v.3.101)^48^ We employed a three-step filtering strategy: 1) Removal of host reads using the species-specific *Drosophila* genome assembled from each corresponding library. 2) Additional filtering using the *Drosophila melanogaster* reference genome (GCF_000001215.4) to eliminate any residual host sequences. 3) Removal of human-derived contaminants using the human reference genome (GCF_000001405.40). This filtering strategy significantly reduced the size of the libraries, and the new *Drosophila*- filtered data sets ranged from 7 to 86 million reads, with an average of 28 ± 19 million sequences. BLAST + (v.2.10.1 +)^49^ was used to query ~ 1000 randomly selected sequences from all libraries against the NCBI nucleotide database (nr). Matches to *D. busckii*, *D. inubila*, *D. virilis*, and *D. hydei* prompted the inclusion of their genomes (GCF_000146045.2, GCF_011750605.1, GCF_004354385.1, GCF_003285735.1, GCF_003285905.1) for further filtering using BMTagger. After the initial clean-up, libraries were reassessed with BLAST + (e-value < 0.001), and additional reads mapping to these genomes (identity > 70%) were removed.

### Sequence profiling of reads

The decontaminated libraries were used to assess bacterial and fungal community composition. Kraken2 (v.2.1.2) was used with a custom database containing 29,635 bacterial and 459 fungal representative genomes from RefSeq (as of October 26, 2022). Bracken (v.2.7.0) was then used to estimate relative taxonomic abundances. Results were filtered using the filter_bracken.out.py script^50^, and relative abundance tables were generated with the combine_bracken_outputs.py script. Data visualisation was performed in R using the *ComplexHeatmap* package (v.2.12.1)^51^ and clustering was conducted using the *vegdist* function from the *Vegan* package (v.2.6–2)^52^.

### Alpha diversity analysis

Bracken-derived relative abundance tables were used in for diversity analysis. Libraries were grouped by *Drosophila* species groups (asiri, guarani, mesophragmatica, repleta, tripunctata), with unclassified species assigned to a “not grouped” category. Chao1 richness was calculated using the *fossil* (v.0.4.0) package^53^ and Shannon diversity index was calculated using the *Vegan* package. Differences in alpha diversity among groups were evaluated using the Kruskal–Wallis test.

### Beta diversity analysis

Beta diversity was assessed using Bray–Curtis and Jaccard dissimilarity metrics, calculated from Bracken abundance tables using the *vegdist* function in *Vegan*(v.2.6–2)^52^. To test for associations between microbiota composition and explanatory variables, permutational multivariate analysis of variance (PERMANOVA) was conducted using the adonis2 function in Vegan with 999 permutations. Separate models were used to evaluate the effects of host species group, geographic origin (province), collection method, most abundant microbial order, and Saccharomycetales relative abundance on community structure. Classical multidimensional scaling (MDS), also known as *principal coordinates analysis*^54^ was performed using the *cmdscales* function from the *stats* package(v.3.6.2)^55^, and visualized with *ggplot2* (*tidyverse* v.3.3.6)^56^.

### De Novo assembly and population genome binning

After additional filtering to remove *Saccharomyces cerevisiae* reads, de novo metagenomic assemblies were performed with MEGAHIT (v.1.2.9)^57^. A minimum contig length of 1000 bp was used to reduce chimerism and contamination^58^. Three binning tools,MaxBin2 (v.2.2.7)^59^, MetaBAT2 (v.2.15)^60^, and CONCOCT (v.1.1.0)^61^ ,were used to minimise bias and improve accuracy. DAS Tool (v1.1.2)^62^ used to consolidate and curate bins based on single-copy gene composition.

The completeness, contamination, and strain heterogeneity were assessed with CheckM (v1.1.3)^63^ which uses lineage-specific single-copy marker genes. The MAGs obtained were classified according to Bowers et al. (2017), as follows: “high-quality” (completeness > 90% and < 5% contamination); “medium quality” (completeness ≥ 50% and < 10% contamination); “low-quality” (completeness < 50% and > 10% contamination). Reads were mapped to MAGs using Bowtie2 (v.2.3.5.1)^64^ to estimate coverage and metagenomic abundances. Bam files were generated and coverage estimated with Samtools (v1.8)^65^.

### Taxonomic annotation and phylogenetic placement

Taxonomic annotation of MAGs was performed using two different tools: the Microbial Genomes Atlas (MIGA)^31^ and the Genome Taxonomy Database Toolkit (GTDB-Tk)^32^. If consensus was not reached, the annotation with the highest ANI or AAI was retained. Gene-level taxonomy was explored using MyTaxaScan (MiGA). Pairwise ANI between high-quality MAGs was estimated with FastANI^66^.

Phylogenetic placement was performed using reference genomes from NCBI (Supplementary Table 4). Gene prediction was done with Prodigal (v 2.6.3)^67^. Essential genes were identified using the HMM.essential.rb script from the Enveomics collection^68^. Genes were aligned using MUSCLE (v3.8)^69^ and concatenated with the script ALN.cat.rb from the enveomics collection. Maximum-likelihood-like trees were calculated using FastTree^70^ with 1000 bootstraps, and the visualisation and annotation were done in iTOL^71^.

### Pathway completeness evaluation

MAGs were annotated using the full set of HMM profiles in the Anvi’o workflow^72^. Metabolic pathways were evaluated using the Kyoto Encyclopedia of Genes and Genomes (KEGG) database with the *anvi-estimate-metabolism* program to assess the completeness of each metabolic module. Functional annotations were assigned by running anvi-run-ncbi-cogs, which associates genes with the Clusters of Orthologous Groups (COGs) database from NCBI. Gene counts within each COG category were computed for all genomes, and differences in COG abundance across taxa were evaluated using ANOVA. Categories with a false discovery rate (FDR) below 0.01 were considered significantly different. A heatmap was generated with the *pheatmap* package, using Euclidean distance for clustering both rows and columns. Row-wise scaling was applied to enhance visualization of variation across functional categories.

### Phylosymbiosis analysis

*Phylosymbiosis* is an analysis that looks for microbial relationships that recapitulate the phylogeny of the host^21^. It consists of comparing distance matrices or between the host phylogeny and a cladogram of its respective microbiota. In this case, we compared the phylogenetic tree made for the twenty-four *Drosophila* species and the resulting cladograms for Bray–Curtis and Jaccard dissimilarity distances of the microbiota. Cladograms were made with the function *upgma* from the *phangorn* package (v.2.9.0)^73^. To compare the similarity between both trees, we used the *TreeDist*^74^ package to calculate the Mutual Clustering Information (MCI) and a Generalized Robinson-Foulds metric, which quantifies the similarity by using the mutual information of two splits within the tree. However, this is only a similarity measure; thus, we calculated a Clustering Information Distance (CID) that was normalised to be bounded between 0 and 1 (= zero similarity). We also generated a phylogenetic tree for MAGs corresponding to *Acetobacter thailandicus* using the same methodology previously described to make a topological comparison to the host phylogenetic tree.

## Supplementary Information

Below is the link to the electronic supplementary material.

Supplementary Material 1

Supplementary Material 2

## Data Availability

The *Drosophila* -filtered metagenomic libraries and high-quality MAGs derived from this study have been deposited in the European Nucleotide Archive (ENA) under Project ID PRJEB70495.
