# Supplementary material for: Bacterial genome reconstruction and community profiling in Neotropical Drosophila
Source: Sci Rep. 2026 Jan 29;16:6601. doi: 10.1038/s41598-026-36282-y (PMC12913781; doi:10.1038/s41598-026-36282-y)
Supplement: Supplementary file 2 — Supplementary Material 2 [file 41598_2026_36282_MOESM2_ESM.pdf]

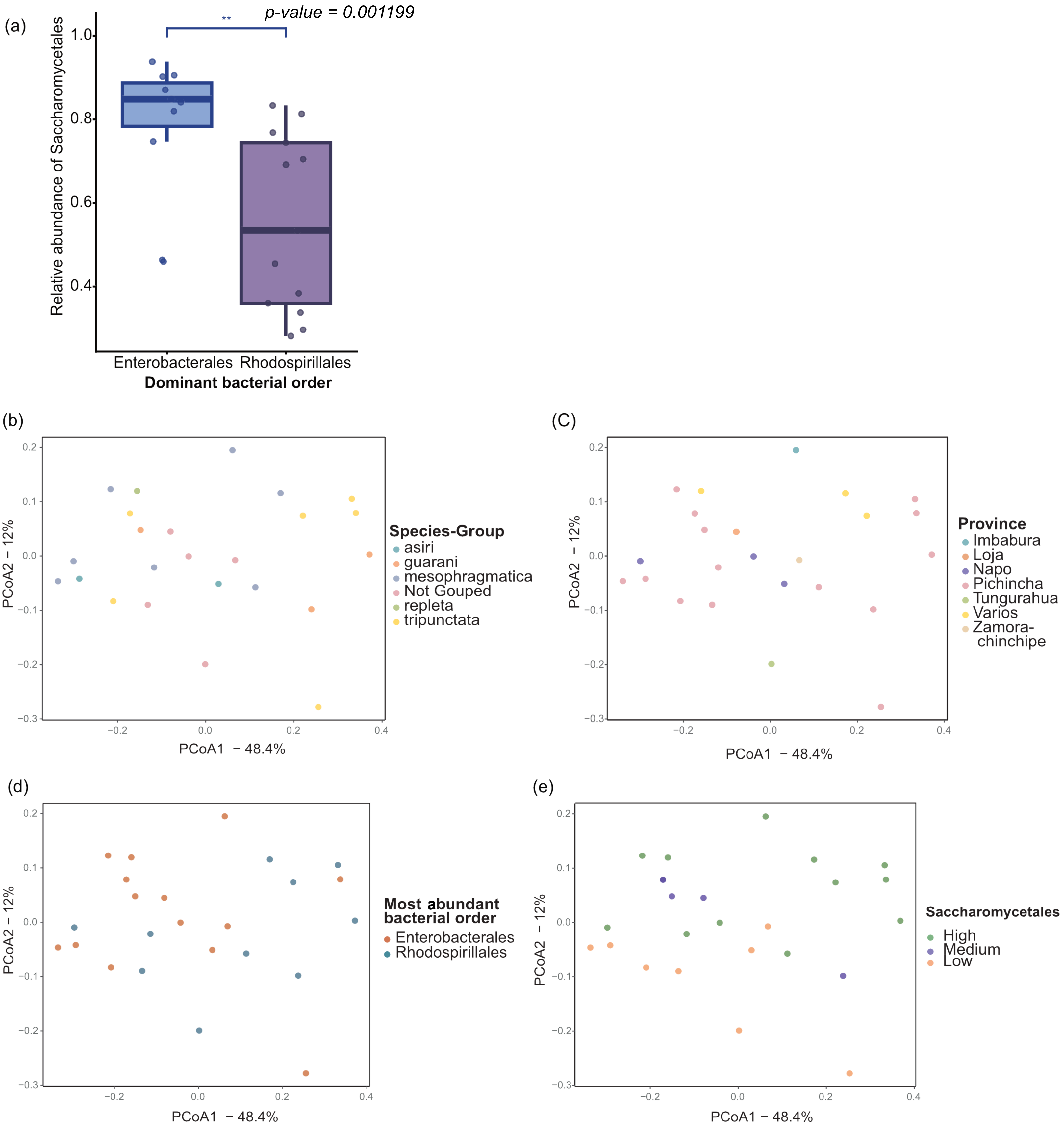


**Supplementary Figure 1.**

(a) Relative abundance of Saccharomycetales across samples grouped by dominant bacterial order. Samples were classified as Enterobacterales-dominated (cornflower blue) or Rhodospirillales-dominated (purple) based on relative abundance. Boxplots show the distribution of Saccharomycetales relative abundance, with points representing individual samples. Statistical significance was assessed using a Wilcoxon rank-sum test. Principal Coordinates Analysis (PCoA) of microbial communities based on the relative abundance of yeast and bacterial reads, excluding *Wolbachia*, using the Jaccard similarity index. Adonis test results are shown for comparisons with significant p-values (*p-value* < 0.001). Each pannel corresponds to: (b) Clustering by Drosophila species group. (c) Collection site of the samples. (d) Dominant bacterial order, defined as taxa represenDng >40% of total bacterial read abundance. (e) Relative abundance of Saccharomycetales yeast: "High" (>60%), "Medium" (30–60%), and "Low" (<30%).

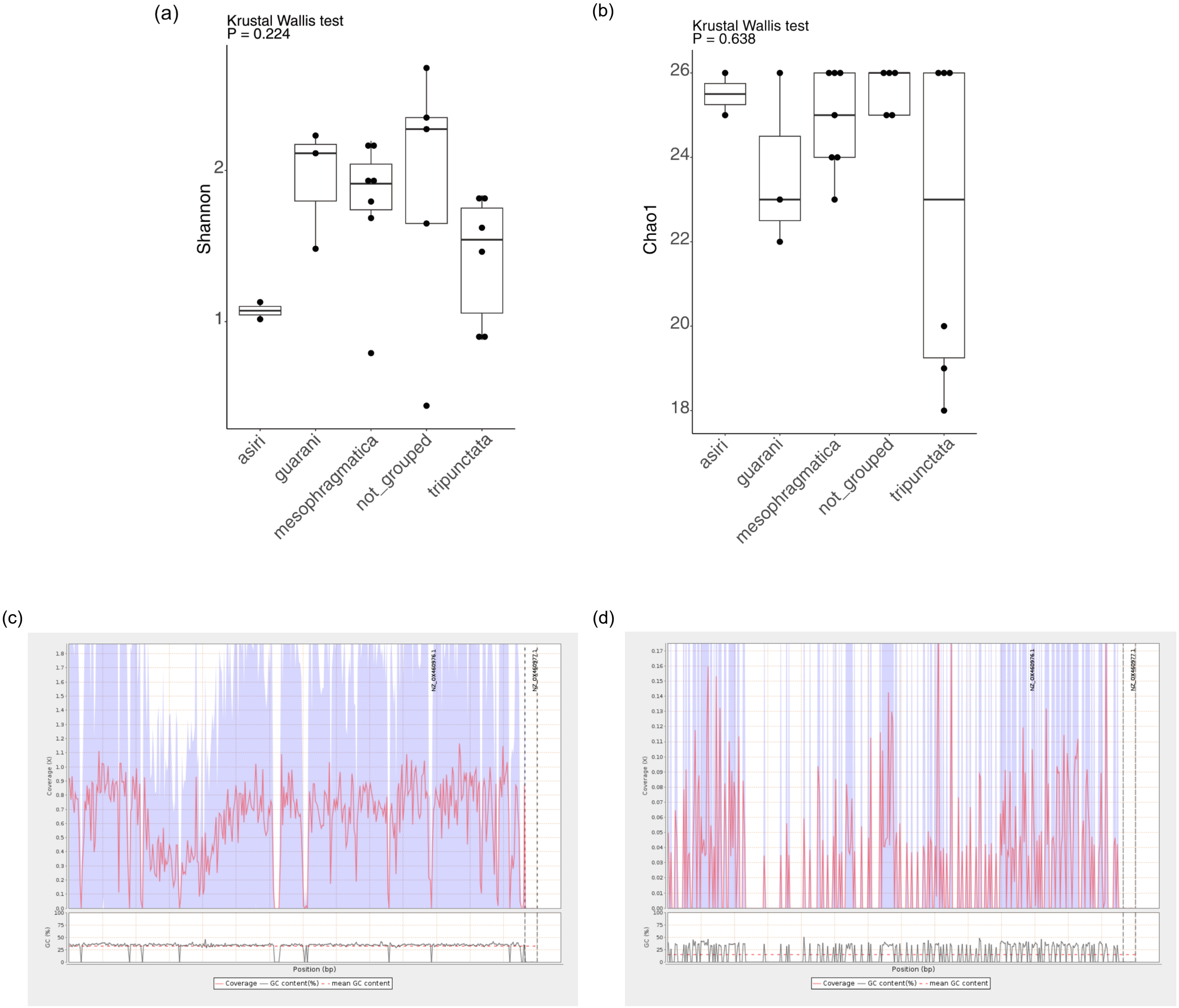


**Supplementary Figure 2.**

Alpha diversity metrics are stratified by *Drosophila* species group. (a) Shannon diversity index; "Not- grouped" indicates samples lacking clear species-group annotation. (b) Chao1 index estimation of richness. Statistical differences were assessed using Kruskal-Wallis's test, with significance set at *p-value* < 0.01.

Read-level versus contig-level mapping highlights challenges in MAG reconstruction, mapping summary Qualimap. (c): Coverage profile obtained by mapping *Carnobacterium*-assigned reads (n = 14,859) from the D. quitensis library to a reference genome of Carnobacterium maltaromaticum (GCF_949790605.1) using Bowtie2 with default parameters, showing high alignment rate (95.7%) and relatively uniform coverage across the genome. (d): Coverage profile obtained by mapping the same reference genome against contigs generated with MEGAHIT, revealing fragmented coverage, reduced depth, and multiple gaps.

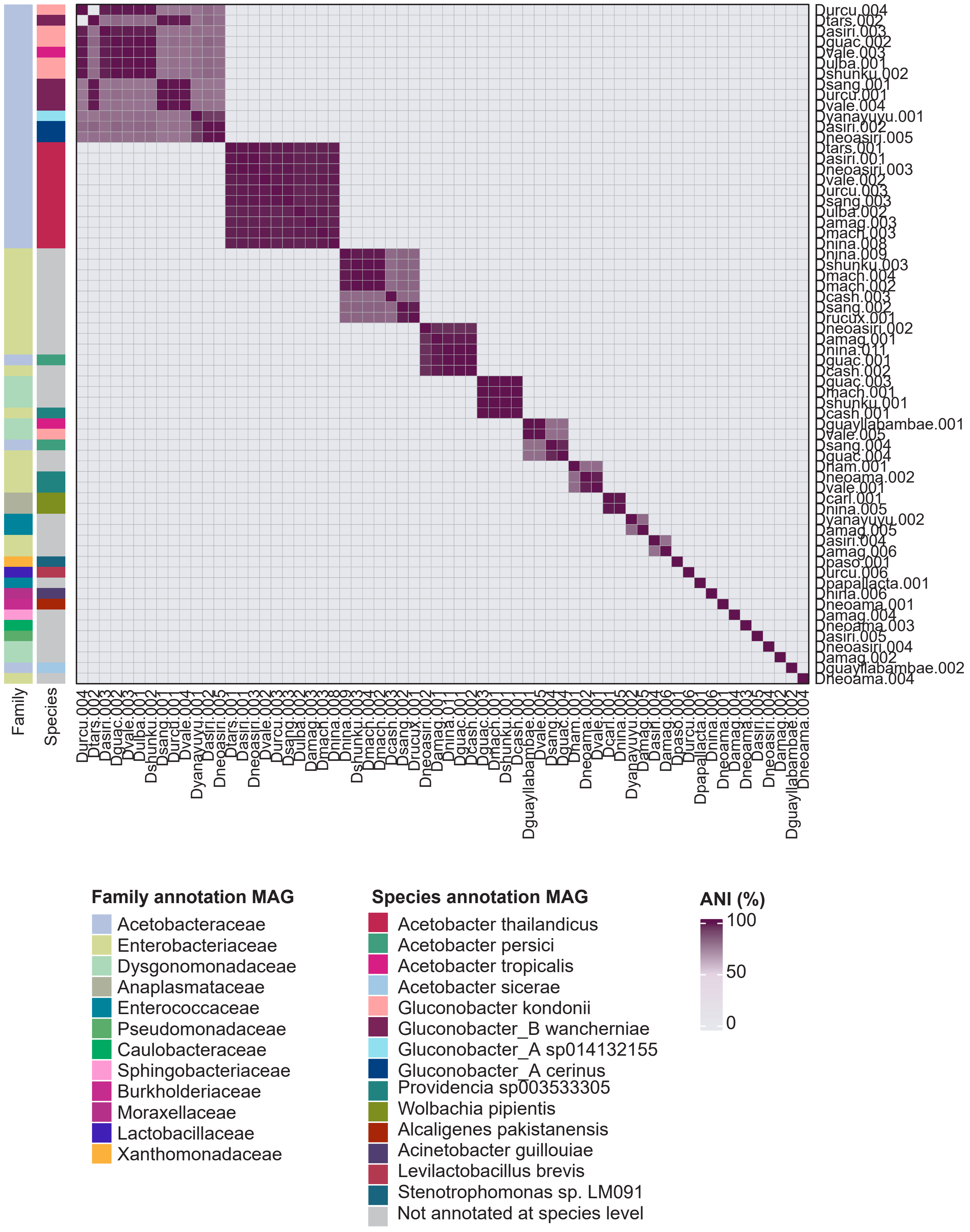


**Supplementary Figure 3.**

Average NucleoDde Identity (ANI) matrix showing pairwise comparisons of high-quality metagenome-assembled genomes (MAGs). ANI values were computed from all-vs-all genome comparisons. Taxonomic labels represent consensus assignments from both the Microbial Genomes Atlas (MiGA) and the Genome Taxonomy Database (GTDB). MAGs are annotated at the species or genus level as available. ANI values below 80% are not displayed.

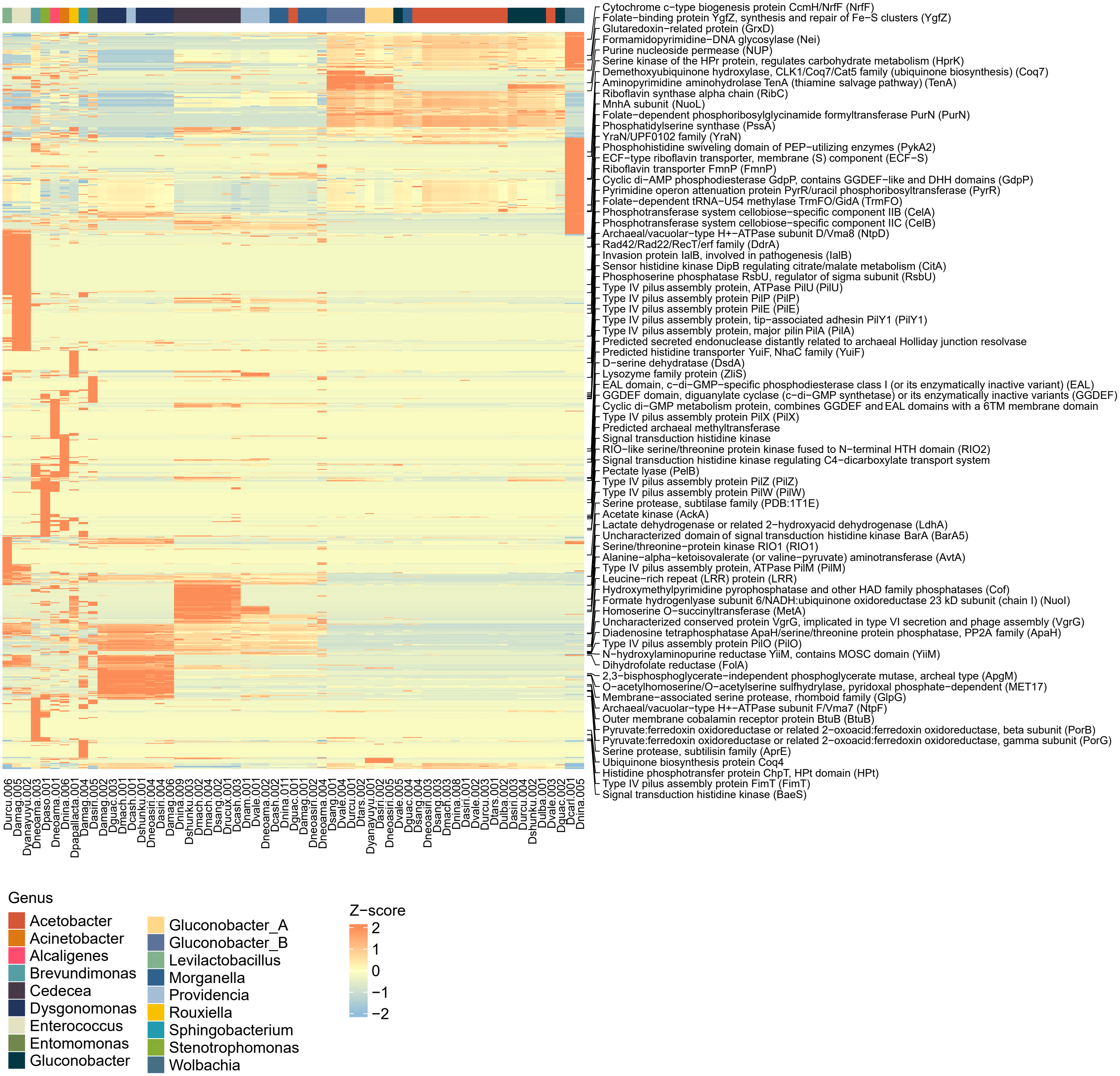


**Supplementary Figure 4.**

Heatmap of relative COG category counts across MAGs. Predicted genes were annotated based on Clusters of Orthologous Groups (COGs) database from NCBI. Gene counts for each category were estimated. Rows correspond to the top 1000 relevant categories. AnnotaDon correspond to funcDons of interest. Values represenDng row-wise z-scores were calculated by centering and scaling COG counts across genomes. Higher scores (red) indicate above-average counts of the COG in each genome, while lower scores (blue) indicate below-average counts.
